# 14-3-3β protein expression in eosinophilic meningitis caused by *Angiostrongylus cantonensis* infection

**DOI:** 10.1186/1756-0500-7-97

**Published:** 2014-02-20

**Authors:** Hung-Chin Tsai, Yen-Lin Huang, Yao-Shen Chen, Chuan-Min Yen, Rachel Tsai, Susan Shin-Jung Lee, Ming-Hong Tai

**Affiliations:** 1 Section of Infectious Diseases, Department of Medicine, Kaohsiung Veterans General Hospital, 386, Ta-Chung 1st Road, Kaohsiung 813, Taiwan; 2 National Yang-Ming University, Taipei, Taiwan; 3 Institute of Biomedical Sciences, National Sun Yat-Sen University, Kaohisung, Taiwan, Republic of China; 4 Department of Parasitology and Graduate Institute of Medicine, Kaohsiung Medical University, Kaohsiung, Taiwan

**Keywords:** *Angiostrongylus cantonensis*, Eosinophilic meningitis, 14-3-3β protein

## Abstract

**Background:**

*Angiostrongylus cantonensis* is a parasite endemic in the Southeast Asian and Pacific regions. Humans are incidentally infected either by eating uncooked intermediate hosts or by consuming vegetables containing the living third-stage larvae. The 14-3-3β protein is a cerebrospinal fluid (CSF) marker of neuronal damage during the development of Creutzfeldt-Jakob disease. In addition, increased 14-3-3β protein is also found in CSF from patients with a variety of neurological disorders. The goal of this study is to determine the roles of serum/CSF14-3-3β protein in patients with eosinophilic meningitis.

**Methods:**

In a cohort study among nine Thai laborers with eosinophilic meningitis due to eating raw snails (*Pomacea canaliculata*), we examined the CSF weekly while patients were still hospitalized and followed up the serum for 6 months. The levels of 14-3-3β protein in CSF were analyzed by western blot and an in-house 14-3-3β enzyme-linked immunosorbent assay (ELISA) measurement was established and tested in an animal model of eosinophilic meningitis.

**Results:**

The elevated 14-3-3β level was detected in the CSF from eight out of nine (81%) patients After 2 weeks of treatment, all patients showed a declined level or cleared of 14-3-3β protein in the CSF. By developing an in-house ELISA for measurement of 14-3-3β protein, it was found that the serum 14-3-3β level was significantly increased in patients during initial visit. . This finding was consistent to the animal experiment result in which there was severe blood brain barrier damage three weeks after infection and increased 14-3-3β protein expression in the CSF and serum by western blot and in house ELISA. After treatment, the serum 14-3-3β level in meningitis patients was rapidly returned to normal threshold. There was a correlation between initial CSF 14-3-3β level with severity of headache (r = 0.692, p = 0.039), CSF pleocytosis (r = 0.807, p = 0.009) and eosinophilia (r = 0.798, p = 0.01) in the CSF of patients with eosinophilic meningitis (Spearman’s correlation test).

**Conclusions:**

The serum 14-3-3β concentrations may constitute a useful marker for blood brain barrier damage severity and follow up in patients with eosinophilic meningitis caused by *A. cantonensis*.

## Background

The major cause of eosinophilic meningitis in the Pacific Islands and Southeast Asia is *Angiostrongylus cantonensis*, also known as the rat lungworm [1-4]. Humans are infected with *A. cantonensis* by ingesting freshwater, terrestrial snails and slugs [5-8]. The major intermediate hosts for *A. cantonensis* in Taiwan are the African giant snail (*Achutina fulica*) and the golden apple snail (*Pomacea canaliculata*) [5,8]. *Pomacea canaliculata* was introduced to Taiwan in 1979 as a food source. It spread widely in paddy fields and drainage ditches and has become an important cause of outbreaks of eosinophilic meningitis [8]. Three outbreaks of eosinophilic meningitis caused by *A. cantonensis* occurred in Kaohsiung, Taiwan, in 1998, 1999 and 2001, respectively [9-11]. Most patients (17/22, 77%) were adult male, Thai laborers who had eaten raw golden apple snails approximately 1 to 3 weeks earlier before hospitalization.

The presence of 14-3-3 protein in cerebrospinal fluid (CSF) has been found to have significant in vivo diagnostic properties in patients with Creutzfeldt-Jakob disease (CJD) [12], and it is thought to result from neuronal disruption and the leakage of brain proteins into the CSF [13]. This protein was found in CSF specimens obtained from patients with various neurological pathologies whose common feature was the presence of some degree of neuronal loss [14-17]. Although its lack of specificity clearly implies substantial limitations in the use of 14-3-3 protein as a specific CJD marker, its value as an indicator of neurological damage could be used to monitor the evolution of different neurological disorders with etiologies that can be otherwise established. Furthermore, the 14-3-3 protein was recently found to be reacted with 31-kDa antigen in *A. cantonensis* infection [18]. Therefore, it was possible to use this protein as a diagnostic tool in *A. cantonensis* infection.

Based on this hypothesis, we studied the dynamic changes of 14-3-3β protein expression in the second outbreak, correlated with CSF findings and established the measurement of 14-3-3β protein expression by enzyme-linked immunosorbent assay (ELISA) and tested in an animal model of eosinophilic meningitis.

## Methods

### Ethics statement

All of the samples of human patients (A to I) were obtained from an outbreak of eosinophilic meningitis in 1999. Those samples were collected at the research laboratory of Infectious Diseases Department, Kaohsiung Veterans General Hospital (KVGH) and the patient’s information was delinked. Institutional review board (IRB) approval to use the samples was obtained from the Commission on Medical Ethics of the Kaohsiung Veterans General Hospital (VGHKS98-CT8-07). All participants were informed about the study procedures and gave their written informed consent initially when the outbreak occurred. Animal studies were carried out in strict accordance with the recommendations from Taiwan’s Animal Protection Act. The protocol was approved by the Animal Committee of the Kaohsiung Veterans General Hospital.

### Study population

A case of eosinophilic meningitis was clinically defined as an acute onset of headache, eosinophil pleocytosis in the blood/CSF, accompanied by at least of one of the following: fever, ataxia, visual disturbances, photophobia, nuchal rigidity, neck pain, hyperesthesias, or paresthesias [10]. All patients who had eaten raw snails within 3 weeks of the outbreak’s onset were included in the study. We recorded demographic information, the date of snails were eaten and the amount ingested, clinical symptoms, and prior parasitic infections. Each patient underwent a physical, neurologic, and ophthalmic examination. The headache intensity was rated on a 4-point scale ranging from none to severe (0 = none, 1+ = mild, 2+ = moderate, 3+ = severe) [10]. The hyperintense basal ganglia lesion on T1-weighted MRI was also graded as a 4-point scale ranging from none to severe by 2 independent radiologists [10]. Spinal taps were performed on all patients. The patients were observed daily during their hospital course. CSF was examined weekly. Peripheral blood was obtained weekly for the first 2 months, every other week for the next 2 months, and monthly thereafter for as long as 6 months. Antibodies to *A. cantonensis* were detected in serum and CSF by a microenzyme-linked immunosorbent assay (ELISA) using young-adult worm antigen, molecular weight 204 kDa purified by monoclonal antibody [19].

### Infection of Balb/C mice with third stage larvae of *A. cantonensis*

Twenty-four Balb/C mice, aged 6–7 weeks, were purchased from the National Laboratory Animal Breeding Research Centre. They were raised and maintained in an air-conditioned animal facility (25 ± 2°C and 50 ± 10% relative humidity). Third-stage larvae of *A. cantonensis* were harvested from infected *Biomphalaria glabrata* after treatment with artificial gastric juice (pepsin, 2 g; concentrated HCl, 7 mL; distilled water, 1 L). Mice were orally infected with 50 *A. cantonensis* L3 via an orogastric tube after slight ether anesthesia and then mice were euthanized every week for 3 consecutive weeks after infection.

### Collection of serum and CSF specimens

Blood samples from experimental mice were collected by a heart puncture under ketamine anesthesia. Serum specimens separated from blood samples after centrifugation at 3500 × *g* (Hermle, Z326K, Germany) for 5 min at 4°C were stored at - 70°C until they were measured.

The skull of the mice was opened after complete bleeding. Careful surgery was conducted in order to avoid blood contamination of the CSF. The brain was removed and washed with 50 μL 0.15 M phosphate buffered saline (PBS). Concurrently, the cerebral ventricles and cranial cavity were washed with 350 μL PBS. The CSF was, thus, harvested with PBS from above, which was then centrifuged in an eppendorf tube at 3000 × *g* (Hermle, Z326K, Germany) for 10 min at 4°C to eliminate cells. The supernatant was stored at -70°C until further use.

### Measurement of permeability of the blood–brain barrier by Evans blue method

Evans blue was used to assess the permeability of the blood–brain barrier to macromolecules. When the blood–brain barrier had been compromised, albumin-bound Evans blue entered the CNS. In brief, a volume of 200 μL of 2% (w/v) solution of Evans blue in PBS was injected into the tail vein of a mouse. One hour later the brain of the mouse was removed after anesthesia with ketamine, which was ground with 1.0 mL PBS in a glass-tissue grinder with a Teflon pestle. The extract was then centrifuged at 18,000 × *g* (Hermle, Z326K, Germany) for 10 min at room temperature. The optical density (OD) of the supernatant was read at 595 nm wavelength using a colorimeter (Thermo scientific multiskan FC, USA). Then the OD values of mice brain supernatant after 1 to 3 weeks infections were compared with the control.

### Measurement of 14-3-3β protein concentrations in CSF/serum by Western blot analysis

CSF/serum aliquots of 100 μL from 9 patients and mice were mixed with 7 volumes of cold methanol, kept at -20°C for 2 h, and then centrifuged at 20,800 *g* for 30 min. The pellet was dissolved in 40 μL of sample buffer (3% SDS, 3% β-mercaptoethanol, 2 mM EDTA, 10% glycerol, and 62.5 mM Tris, pH, 6.8) and boiled for 5 min. For each sample, 10 μL (the equivalent of 25 μL of CSF), 5 mL, and 1.25 mL of sample buffer/well were loaded onto a 13% polyacrylamide gel and transferred to polyvinyliden difluoride membranes (Immobilon P; Millipore). Membranes were incubated with anti 14-3-3β polyclonal rabbit IgG (Santa Cruz Biotechnology) at a 1:500 dilution and revealed with anti-rabbit horseradish peroxidase IgG (Amersham) at a 1:3000 dilution. The blots were developed using an enhanced chemiluminescent system (Amersham). Densitometric values for each sample were obtained with a computer-assisted laser scanner (GS-710 Calibrated Imaging Densitometry; BioRad), after correction for background. The total amount of 14-3-3 protein as quantified from each diluted and undiluted CSF sample was expressed in arbitrary units [19]. The human control group (n = 9), matched for age and gender, consisted of patients with headache or altered consciousness who underwent lumbar puncture to exclude meningitis. CSF samples were centrifuged and the supernatants were frozen at -80°C until assayed.

### Generation of recombinant 14-3-3β

Recombinant human 14-3-3β protein was purified from *E. coli* for antibodies generation as previously described [20]. The human 14-3-3β cDNA was amplified from a human fetal brain cDNA library (Stratagene, La Jolla, CA) using the polymerase chain reaction (PCR). The PCR primers used to clone the human 14-3-3β cDNA were designed based on the 14-3-3β sequence in the Gen-Bank database (accession number, NM_003404.3; forward primer, 5′- cgcggatccatgacaatggataaaagtgagctg -3′; reverse primer, 5′- ggcgaattcttagttctctccctccccagc-3′). After DNA sequencing analysis, the PCR-amplified 14-3-3β cDNA was subcloned into the *EcoR*I and *BamH*I sites of the pET28a vector (Novagen, Madison, WI) and transformed into BL-21 cells (DE3, pLysS; Novagen). After induction, the 6x-histidine-tagged 14-3-3β protein was purified on an NTA-agarose affinity column (Qiagen, Hilden, Germany) and desalted on a G25 Sephadex column (Amersham Pharmacia, Little Chalfont, United Kingdom). The recombinant protein was passed through Detoxi-Gel (Pierce Biotechnology, Rockford, IL) to minimize contamination by endotoxin. The 14-3-3β antibodies were raised by periodic injection of recombinant 14-3-3β protein into rabbits. The serum was collected from immunized rabbits and analyzed using Western blot analysis.

### Production of anti-human 14-3-3β polyclonal antisera

#### Primary immunization

Purified recombinant 14-3-3β protein in PBS (500 μg in 500 μl) is mixed with complete Freunds adjuvant in a three-way stopcock until mixture becomes emulsified. The mixture is then transferred to a 3 ml 24-gauge syringe and was injected subcutaneously into adult New Zealand white rabbits (2 to 5 kg body weight) under restrain. Two weeks after the primary immunization, the rabbits were then boosted with recombinant 14-3-3β protein mixed with incomplete Freunds adjuvant at 2-week intervals for a total of nine boosts.

#### Blood serum preparation

Blood was collected from the ear marginal veins of restrained rabbits before immunization (pre-immune serum) and after subsequent boosts for a total of six batches. About 50 ml of blood was collected during each interval. After removing blood clots, the serum samples were placed overnight in 4°C and centrifuged 10 minutes at 5,000 g to remove red blood cell pellets and other cell debris. The samples were stored at -70°C prior to purification.

#### Antibody purification

Serum samples containing 14-3-3β antibodies were purified using Protein A column (Pharmacia Biotech) and quantified with Coomassie Plus Bradford assay kit (Pierce, #23236). Antibody efficacy was checked with Western blot analysis using recombinant 14-3-3β protein at concentration of 1, 0.1 and 0.01ug/ml.

### 14-3-3β enzyme-linked immunosorbent assay (ELISA)

The in-house 14-3-3β ELISA consisted of rabbit anti-14-3-3β polyclonal antibody as the capture antibody and mouse anti-14-3-3β IgG conjugated with horse-radish peroxidase (Santa Cruz Biotechnologies Inc.; sc-1657 HRP (H-8), 200 μg/ml) as the detection antibody, with the purified recombinant 14-3-3β protein as standard. Briefly, 96-well plate were coated with 50 μl per well of diluted capture antibody (2.5 μg/ml) overnight at 4°C. After washing with buffer containing 0.05% Tween 20 in PBS, wells were blocked with buffer containing 0.05% Tween 20, 0.1% BSA, 5% sucrose in PBS at room temperature for 30 min. The CSF/serum samples (50 μl) or standards were applied to wells and incubated at room temperature for 1 hour. After wash, the wells were incubated with detection antibody (mouse anti-14-3-3β, 1 μg/ml) at room temperature for 1 hour. Finally, 100 μl of TMB substrate solutions were added and reacted at room temperature for 20 min. After adding 50 μl of 2 N H_2_SO_4_, the optical density in each well was measured at 450 nm using an ELISA reader.

### Statistical analysis

The relation between the amount of 14-3-3β protein in CSF, laboratory abnormalities, and clinical severity and MRI findings were analyzed with Spearman’s correlation coefficient test. Wilcoxon signed-rank test was used to compare the change of 14-3-3β protein in every week of lumbar puncture. Mann Whitney U test was used to compare the changes of 14-3-3β protein or Evans blue in third week relative to the controls. A *P* value <0.05 was considered statistically significant.

## Results

In the current studies, all nine patients were young Thai men. The source of epidemic was ingestion of raw snails seasoned with lemon juice and red pepper. Antibodies to *A. cantonensis* were detected at the time of admission in the serum of 9 (100%) patients and in the cerebrospinal fluid of 4 (44%) patients. Patients in this outbreak in 1999 only received supportive therapy (acetaminophen and naproxen), lumbar puncture was done when clinically indicated. None received steroid treatment. All of the nine patients received a total of 23 lumbar punctures. Patient F received a total of 3 lumbar punctures. Patient C and I have 4 lumbar punctures due to recurrent symptoms. Headache, stiff neck, transient right facial palsy, ataxia, and diplopia recurred in patient C 2 weeks after last admission; he was treated with intravenous glycerol for 7 days and recovered completely. Vomiting and headache developed in patient I 3 weeks after admission and a spinal tap revealed an elevated opening pressure of 240 mm H_2_O, a white cell count of 1110 × 10^3^ cells per μL with 74% eosinophils. He recovered without treatment in about a week.

**Figure 1:**
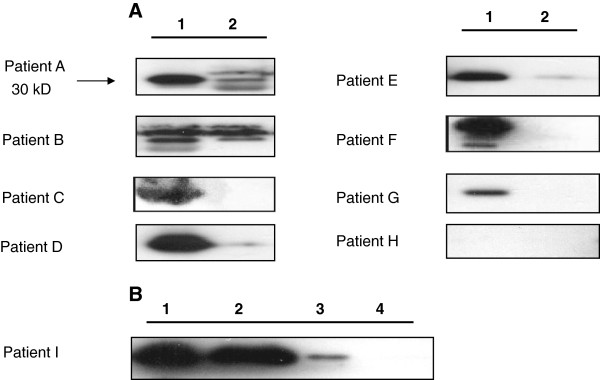
**Dynamic changes of 14-3-3β protein expression in 9 patients with eosinophilic meningitis. A)** Expression of 14-3-3β protein in the CSF of patients with eosinophilic meningitis after visits for treatment. Numbers indicate time intervals of lumbar punctures (units are in weeks). Eight out of nine patients show detectable level of 14-3-3β protein on admission at the hospital. Lumbar punctures performed on one week after admission show decreased expression in the CSF (Wilcoxon signed-rank test, p = 0.025). **B)** Expression profile of 14-3-3β protein in patient I. It showed clearance of CSF 14-3-3β protein 3 weeks after presentation (**1**: admission; **2**: One week after presentation; **3**: Two weeks after presentation; **4**: Three weeks after presentation).

The Western blot analysis for 14-3-3β protein in CSF was shown in Figure 1. Eight out of nine patients had a Detectable 14-3-3β protein in CSF at admission. The initial 14-3-3β protein amount in CSF, severity of headache, laboratory abnormalities, and findings in the brain MRI scan are listed for each patient in Table 1. There was a correlation between initial CSF 14-3-3β level with severity of headache (r = 0.692, p = 0.039), CSF pleocytosis (r = 0.807, p = 0.009), and CSF eosinophilia (r = 0.798, p = 0.01) in patients with eosinophilic meningitis (Spearman’s correlation test). Wilcoxon signed-rank test was used to compare the change of 14-3-3β protein in each lumbar puncture and it revealed there was a significant decrease of 14-3-3 protein amounts in the 2^nd^ week (one week) after admission (Wilcoxon signed-rank test, p = 0.025). Patient C and I had only mild elevation of 14-3-3 protein when recurrence of symptoms. Nine patients with a diagnosis of sepsis underwent lumbar puncture for excluding meningitis as control group all showed negative 14-3-3β protein expression in CSF and serum.

**Table 1:** **Initial 14-3-3β protein amount and laboratory and magnetic resonance imaging scan findings in 9 cases of eosinophilic meningitis caused by** ***A. cantonensis***

	**Blood**		**Cerebrospinal fluid**						
**Patient**	**White blood cell count**	**Eosinophil**	**Protein**	**Glucose**	**White blood cell count**	**Eosinophil**	**14-3-3β**	**Headache**	
	**(×10**^**3**^**/μL)**	**(%)**	**(mg/dL)**	**(mg/dL)**	**(×10**^**3**^**/μL)**	**(%)**	**Protein(DU)**	**severity**	**MRI**
A	8210	8	67	75	1	0	6.8	1	ME, 1+
B	7570	10	49	60	0	0	1.1	1	ME, 1+
C	10500	36	154	36	1270	36	0	3	ME, 3+
D	6990	16	36	66	139	6	9.8	1	ME, 2+
E	5920	7	95	72	9	0	0	1	ME, 1+
F	9480	16	347	45	1660	6	7.8	3	ME, 2+
G	6490	20	50	71	1	0	0	1	ME, 1+
H	8510	2	27	70	0	0	0	2	ME, 2+
I	13270	29	201	59	1390	17	5.1	3	ME, 2+

ME: meningeal enhancement; MRI: magnetic resonance imaging; Severity of headache is graded as follows: 0 none, 1+ mild, 2+ moderate, 3+ severe. The MRI grading is as follows: 0 none, 1+ mild, 2+ moderate, 3+ high signal intensity in bilateral globus pallidus on T1 imaging. All of the 9 patients received only supportive treatment (acetaminophen and naproxen), lumbar puncture was done when clinically indicated.

### Detection of 14-3-3β levels in patient CSF/Serum samples

**Figure 2:**
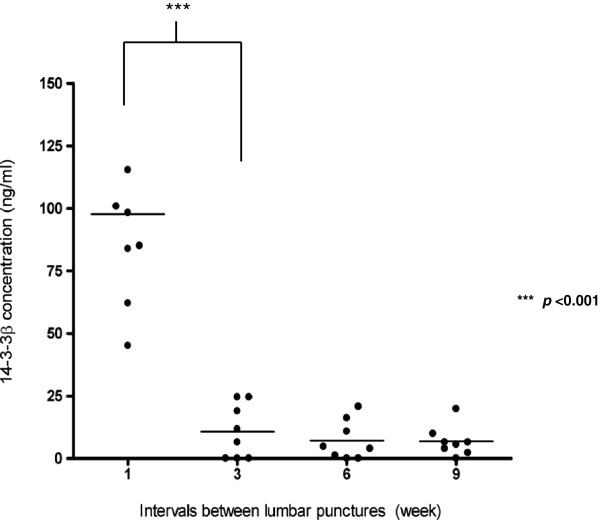
**Concentration of 14-3-3β proteins in the patient serum samples.** Each weekly interval contain the collective serum samples taken from the nine patients at that particular time period. ELISA analysis performed on the serum samples showed significant decrease in 14-3-3β protein levels 2 weeks after admission (Wilcoxon signed-rank test, p = 0.012). **1**: admission; **3**: Two weeks after presentation; **6**: Five weeks after presentation; **9**: Eight weeks after presentation). One patient had no follow up data.

Using polyclonal antibodies constructed from rabbit immunized with recombinant 14-3-3β antigen, we have established a procedure to detect 14-3-3β levels in CSF and serum using ELISA. We have found that the level of 14-3-3β protein in the patient serum decreased over an eight week period after on admission to the hospital (Figure 2). There was a near four-fold reduction of the protein at about two weeks after the first lumbar puncture (Wilcoxon signed-rank test, p = 0.012). However, we did not observe significant changes in the CSF samples due to the inadequate samples numbers and CSF volumes.

### Permeability of the blood–brain barrier by Evans blue method in the animal study

**Figure 3:**
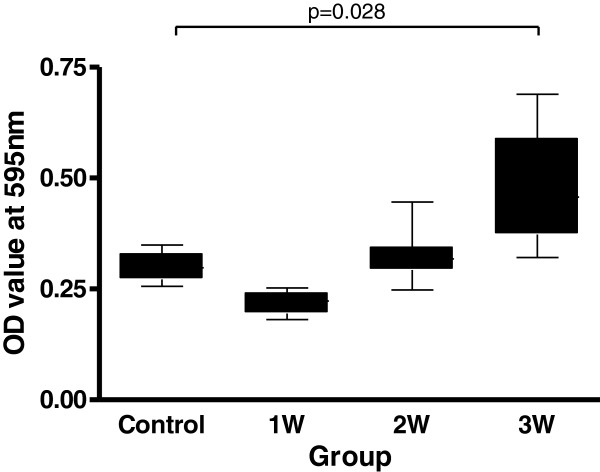
**Dynamic changes of Evans blue amounts in mice with eosinophilic meningitis caused by *A. cantonensis *infection.** The amount of Evans blue in the mice brain showed significantly increase 3 weeks after infection compared to those of uninfected mice (p = 0.028).

Uninfected control mice with the optical density of the extract of the brain measured at 595 nm were similar with the blank control. In contrast, the brain of infected mice showed varying degrees of staining by Evans blue after infection. The amount of Evans blue in the mice brain showed significantly increase 3 weeks after infection compared to those of uninfected mice (p = 0.028) Figure 3.

### Western blot analysis of 14-3-3β protein in the CSF/serum of mice

**Figure 4:**
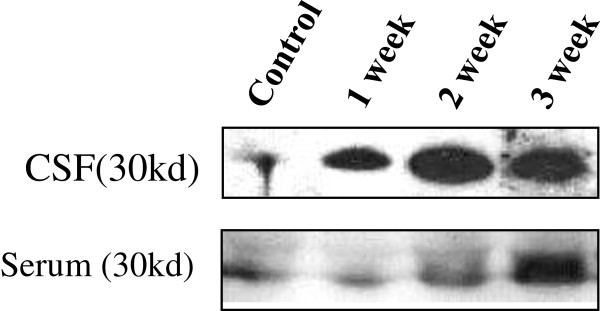
**Expression of 14-3-3β protein in the serum and CSF of mice with eosinophilic meningitis by the western blot assay.** There was a significant increase of 14-3-3β protein amounts of serum/CSF in the 3^rd^ week after infection compared to the controls.

The Western blot analysis for 14-3-3β protein expression in the CSF/serum of mice was shown in Figure 4. There was a significant increase of 14-3-3β protein amounts of serum/CSF in the 3^rd^ week after infection compared to control. This finding was consistent the severity of blood brain barrier damage as evidence by the Evans Blue assay result.

### Detection of 14-3-3β levels in mice CSF/Serum samples by in house ELISA

**Figure 5:**
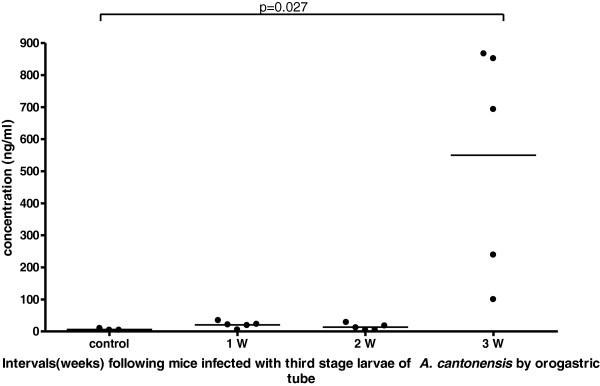
The levels of 14-3-3β protein in the CSF increased over a three week period after infection and the data was shown by an in house ELISA.

**Figure 6:**
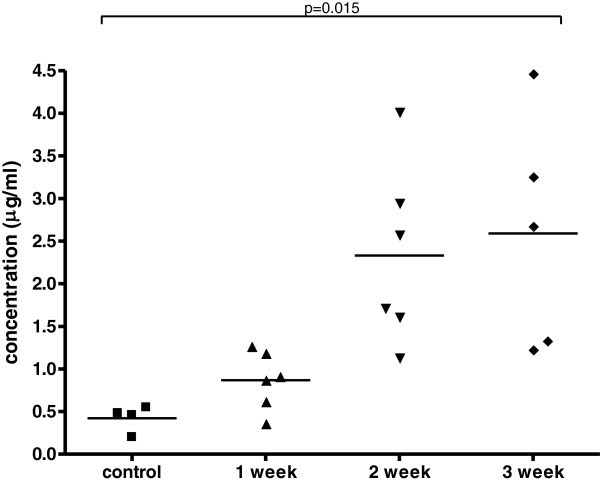
The levels of 14-3-3β protein in the serum increased over a three week period after infection and the data was shown by an in house ELISA.

Using polyclonal antibodies constructed from rabbit immunized with recombinant 14-3-3β antigen, we have established a procedure to detect 14-3-3β levels in CSF and serum using ELISA. We found that the level of 14-3-3β protein in the CSF/serum increased over a three week period after infection Figures 5 and 6.

## Discussion

We found that the amounts of CSF 14-3-3β protein were increased in our patients at presentation. After 2 weeks of treatment, all patients showed a declined level or cleared of 14-3-3β protein in the CSF. We also found that the serum 14-3-3β level was significantly increased in patients during initial visit by our in house ELISA. This probably indicated that the leakage of 14-3-3β from the brain because of neuronal damage and blood brain barrier dysfunction. This finding was consistent to the animal experiment result in which there was severe blood brain barrier damage three weeks after infection and increased 14-3-3β protein expression in the CSF and serum by western blot and in house ELISA.

The 14-3-3 proteins belong to a family of acidic, dimeric proteins that are expressed in all eukaryotic cells. This family of highly conserved proteins plays crucial roles in regulating multiple cellular processes including the maintenance of cell cycle checkpoints and DNA repair, the prevention of apoptosis, the onset of cell differentiation and senescence, and the coordination of cell adhesion and motility [21]. The highest tissue concentration of 14-3-3 proteins is found in the brain, comprising about 1% of its total soluble protein [22] and being present in the cytoplasmic compartments, plasma membrane and in intracellular organelles [23]. In addition to their possible role in neuronal function, 14-3-3 proteins have attracted much recent interest owing to their possible involvement in the pathophysiology of various neurological disorders [14-17,24]. However, there had been no reports in the literature that deal with the association of 14-3-3 protein and eosinophilic meningitis caused by *A. cantonensis.* In our cohort study, nine patients with eosinophilic meningitis, in addition to the commonly tested CSF parameters, were serially measured the CSF concentrations of 14-3-3β proteins. Most of the patients (8/9, 90%) whom we investigated had a positive test result at admission, and all of those who recovered cleared 14-3-3β protein from CSF 2–3 weeks after hospitalization. This dynamic change of 14-3-3β protein in CSF was consistent to the clinical course of recovery in our patients who received 2 weeks of treatment of painkiller and serial spinal tapping [9]. If 14-3-3β proteins in CSF were used to monitor the evolution of a reversible condition, a marker of tissue damage must quickly clear once the damaging process has subsided, as in our cases.

We found a fairly good sensitivity of the 14-3-3β test. This result, however, had weak significance because they were based on only 9 patients with eosinophilic meningitis. But more importantly, we examined a homogenous population, regularly monitored CSF dynamic change and excluded patients with other neurological conditions such as other infectious encephalitis, dementia, brain tumor, metabolic encephalopathy, neuropathy and Creutzfeldt-Jakob disease, that would otherwise cause elevation of 14-3-3β protein in CSF [12-17]. It has been shown that inappropriate handling and storage of CSF samples may alter the results of the test [13]. In our study, lumbar puncture techniques were optimal and identical in each case. The possible reason explaining the only one negative test result could be inappropriate handling and storage of CSF, technical problem, and threshold of 14-3-3 protein detection. Another most importantly reason was that the patient (patient H) was diagnosed as *A. cantonensis* infection only based on the exposure history and positive serum serology. He had no peripheral eosinophilia or CSF abnormalities which are commonly seen in patients with eosinophilic meningitis caused by *A. cantonensis* infection. Finally, the time interval between disease onset and the 14-3-3β test was similar in the eosinophilic meningitis group and control group (13 vs.12 days, respectively), suggesting that disease duration alone cannot account for the dynamic change of 14-3-3β protein in CSF. Taken together, our data suggest that, when performed as a screening test in a patient with eosinophilic meningitis, a positive 14-3-3β test result may rule in this diagnosis. Another matter of concern comes from the questionable reliability of the 14-3-3β test, because in two of our patients with relapse (patient C, I), the result was found to be first positive and then only weakly positive when relapse occurred. This situation does not seem to be unique because it has already been observed by others in four non-CJD patients who were initially found positive and then negative 2 weeks later [25]. Possible explanations include instability of the 14-3-3β protein in the sample over time, or clinical recurrence of symptoms/signs which did not cause ongoing neuronal damage. To solve these issues, further controlled, multicenter studies assessing the validity of the 14-3-3β test are needed.

Identification of 14-3-3 proteins in CSF of patients afflicted with a wide range of neuro-degenerative diseases has been well documented [17,26], however there is few literatures which discuss the presence of such protein in the serum. In the current study, we presented the in-house ELISA as another diagnostic mean to validate the western blot analysis of 14-3-3β proteins in the CSF and serums samples collected. We found that there was a significant decrease in serum 14-3-3β level from onset of meningitis to two weeks after admission to the hospital, and so on until 8^th^ week. However, there were no significant changes in CSF 14-3-3β levels between each lumbar puncture intervals as opposed to results from western blot. It is likely that limited CSF sample numbers and inadequate CSF volumes for ELISA contributed to such discrepancy, as the results from ELISA would be less accurate and sensitive if there weren’t enough samples available for analysis.

The presence of 14-3-3β proteins in serum samples may be the consequences of disruption of the blood brain barrier caused by the meningitis, as it was shown that infection by the parasite may induce up-regulation of inflammatory agents such as MMP-9 [27], and contributes to the degradation of meningeal blood vessel membranes. This change in the cerebral vasculature could account for the leakage of 14-3-3β, primarily a CNS protein, into the peripheral circulation. In terms of clinical application, the ability to detect 14-3-3β proteins in serum samples may yield potential diagnostic values such ease of sample acquisition, reduced discomfort for patients undergoing diagnosis and increased efficiency in sample handling/detection. However, the current technique may require additional refinements to increase its sensitivity and accuracy before it could be implemented as an alternative diagnostic tool of neurodegenerative disease.

## Conclusion

The current study found that patients with eosinophilic meningitis and presence of 14-3-3β proteins in CSF tended to have more severe CSF laboratory abnormalities. The decline of 14-3-3β proteins 2–3 weeks after admission was consistent with the idea that 14-3-3 protein levels are associated with the pace of neuronal injury.

## Competing interests

The authors declare that they have no competing interests.

## Authors’ contributions

Conceived and designed the experiments: HCT, YSC, and MHT. Performed the experiments: YLH, and RT. Analyzed the data: CMY and SSJL. Wrote the paper: HCT, YSC, and MHT. All authors read and approved the final manuscript.
